# Phenolics from *Rhagadiolus stellatus* (Asteraceae, Cichorieae)

**DOI:** 10.3797/scipharm.1011-12

**Published:** 2011-02-07

**Authors:** Romana Krimplstätter, Benjamin Ma, Renate Spitaler, Ernst Ellmerer, Christian Zidorn

**Affiliations:** 1 Institut für Pharmazie, Universität Innsbruck, Josef-Moeller-Haus, Innrain 52, A-6020 Innsbruck, Austria; 2 Institut für Lebensmitteluntersuchung Innsbruck, Österreichische Agentur für Gesundheit und Ernährungssicherheit, Technikerstraße 70, A-6020 Innsbruck, Austria; 3 Institut für Organische Chemie, Universität Innsbruck, Innrain 52a, A-6020 Innsbruck, Austria

**Keywords:** *Rhagadiolus stellatus* Gaertn., Asteraceae, Cichorieae, Flavonoids, Chemosystematics

## Abstract

*Rhagadiolus stellatus* Gaertn., a Mediterranean member of the Cichorieae tribe of the Asteraceae family used as a food plant, was analyzed for its spectrum of phenolic compounds. Kaempferol 3-*O*-β-glucoside **1**, kaempferol 3-*O*-β-rutinoside (nicotiflorin) **2**, quercetin 3-*O*-β-glucoside **3**, and luteolin **4** were isolated from the *n*-butanol layer of a methanolic extract of whole plants of *Rh. stellatus* of Spanish origin by repeated Sephadex LH-20 column chromatography. Structures were determined based on NMR and MS data as well as by comparison with literature data. Additionally, chlorogenic acid **5** and 3,5-dicaffeoylquinic acid **6** were detected by HPLC/DAD and HPLC/MS. Chemosystematic implications of the presented findings are discussed in comparison with other members of the Cichorieae tribe.

## Introduction

*Rhagadiolus stellatus* (L.) Gaertn. is one of two species of the genus *Rhagadiolus*. According to recent molecular studies *Rhagadiolus* is closest related to the genus *Lapsana* and both genera cluster within the genus *Crepis* s.l. [1]. *Rhagadiolus stellatus* is a herb of up to 50 cm height, with small flowering heads composed of yellow ligulate flowers. The achenes are narrowly cylindrical and bear no pappus. The outer achenes are long-persistent and form a characteristic radiating infructescence, as also indicated in the specific epithet. The natural distribution area of *Rh. stellatus* encompasses Southern Europe, Northern Africa, the Macaronesian Archipelago, and the West and Southwest of Asia [2, 3]. In the only phytochemical investigation of *Rh. stellatus* so far, quercetin was found to be the major aglycon in leaves after hydrolysis of the genuine flavonoids [4].

## Results and Discussion

**Fig. 1.**
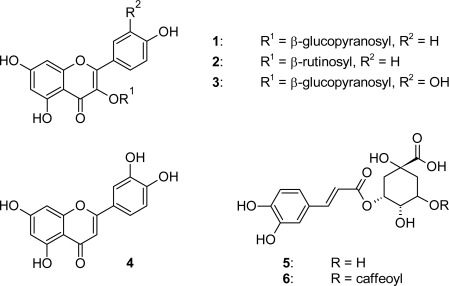
Structures of phenolics isolated from and detected in *Rhagadiolus stellatus*.

Flavonoids (Fig. 1) kaempferol 3-*O*-β-glucoside **1**, kaempferol 3-*O*-β-rutinoside (nicotiflorin) **2**, quercetin 3-*O*-β-glucoside **3**, and luteolin **4** were identified based on NMR and MS data as well as by comparison with literature data of the above and related compounds [5–12]. Phenolic acids chlorogenic acid **5** and 3,5-dicaffeoylquinic acid **6** (Fig. 1) were detected by HPLC/DAD and HPLC/MS in comparison with authentic reference compounds using an established system [13].

Chlorogenic acid **5** and 3,5-dicaffeoylquinic acid **6** occur ubiquitously in the Asteraceae. Luteolin **4** is a very common flavonoid in the Cichorieae and has also been reported from both *Crepis* and *Lapsana*, which are closely related to *Rhagadiolus* [1, 14]. Quercetin 3-*O*-glucoside **3** was also reported from aerial parts of *Lapsana communis* L. but has not yet been found in *Crepis* [14]. In contrast, kaempferol 3-*O*-derivatives **1** and **2** are rather rare flavonoids in the Cichorieae tribe. Kaempferol 3-*O*-β-glucoside **1** has only been reported from the genus *Cichorium* (detected in leaves of four species), aerial parts of *Lactuca tatarica* C.A.Mey., and in whole plants of five species of *Stephanomeria*. Kaempferol 3-*O*-β-rutinoside **2** has only been reported from aerial parts of *Pinaropappus roseus* Less. and *Scolymus hispanicus* L. [14].

A deeper chemosystematic interpretation of the above findings is difficult because of the poor or missing phytochemical data for most of the related genera. Phenolics of many species of *Crepis* were reported recently but these data are of limited value in the present context because this study was focused on flowering heads, only [15]. Nonetheless, the data presented here suggest that *Rhagadiolus* is not only morphologically but also chemically (prevalence of flavonols) differentiated from the genus *Crepis* (prevalence of flavones) and thus the decision of Enke and Gemeinholzer [1] to keep *Rhagadiolus* as a genus separate from *Crepis* is compatible with the available phytochemical data.

## Experimental

### Plant material

*Rh. stellatus* was collected in April 2009 between Vélez Rubio and Santa Maria de Nieva/Almeria/Andalucia/Spain; N 37°37′26″; W 02°00′53″; alt.: 890 m. Voucher specimens are deposited in the herbarium of the Institut für Botanik, Universität Innsbruck, Austria (voucher codes: IB-33270) and in the private herbarium of CZ (CZ-20090417A-2).

### Natural product isolation and identification

Air-dried, ground whole plants (719 g) of *Rh. stellatus* were exhaustively macerated with MeOH to yield 101 g of crude extract after evaporation of the solvent *in vacuo*. The crude extract was re-dissolved in a mixture of MeOH and H_2_O (1/2, v/v) and successively partitioned with petrol ether, EtOAc, and *n*-BuOH. The BuOH layer was brought to dryness *in vacuo* to yield 11.8 g of residue.

Kaempferol 3-*O*-β-glucoside **1** (22.4 mg), kaempferol 3-*O*-β-rutinoside (nicotiflorin) **2** (9.0 mg), quercetin 3-*O*-β-glucoside **3** (62.6 mg), and luteolin **4** (3.8 mg) were isolated from the BuOH layer of a MeOH extract of whole plants of *Rh. stellatus* by repeated Sephadex LH-20 column chromatography using a mixture of MeOH, (CH_3_)_2_CO, and H_2_O (3/1/1, v/v/v) as mobile phase. Chlorogenic acid **5** and 3,5-dicaffeoylquinic acid **6** were detected by HPLC/DAD and HPLC/MS in comparison with authentic reference compounds using the methodology described by Fusani and Zidorn in 2010 [13].

NMR spectra were measured at 300 MHz (^1^H NMR) and 75 MHz (^13^C NMR), respectively. Spectra of compounds **1**, **2**, and **4** were recorded in CD_3_OD and referenced to solvent residual and solvent signals at 3.31 ppm (^1^H NMR) and 49.0 ppm (^13^C NMR), respectively. Spectra of compound **3** were recorded in DMSO-*d*_6_ and referenced to solvent residual and solvent signals at 2.50 ppm (^1^H NMR) and 39.5 ppm (^13^C NMR), respectively.

## Supporting Information



## Supporting Information

A scan of a voucher specimen (Fig. S1) is available in the online version (Format: PDF, Size: ca. 0.6 MB): http://dx.doi.org/10.3797/scipharm.1011-12
