# Supplementary material for: Phenolics from Rhagadiolus stellatus (Asteraceae, Cichorieae)
Source: Sci Pharm. 2011 Feb 7;79(1):175–9. doi: 10.3797/scipharm.1011-12 (PMC3097504; doi:10.3797/scipharm.1011-12)
Supplement: Supplementary file 1 [file Scipharm_2011_79_175_supporting_information.pdf]

Scientia Pharmaceutica www.scipharm.at

Open Access

Supporting Information to

Phenolics from *Rhagadiolus stellatus* (Asteraceae, Cichorieae)

Romana Krimplstätter, Benjamin Ma, Renate Spitaler, Ernst Ellmerer, Christian Zidorn

Published in Sci Pharm. 2011; 79: 175–179 doi:10.3797/scipharm.1011-12

Available from: http://dx.doi.org/10.3797/scipharm.1011-12

© Krimplstätter *et al.*; licensee Österreichische Apotheker-Verlagsgesellschaft m. b. H., Vienna, Austria.

This is an Open Access article distributed under the terms of the Creative Commons Attribution License (http://creativecommons.org/licenses/by/3.0/), which permits unrestricted use, distribution, and reproduction in any medium, provided the original work is properly cited.

Table of Contents

Fig. S1. Voucher of Rhagadiolus stellatus (L.) Gaertn.

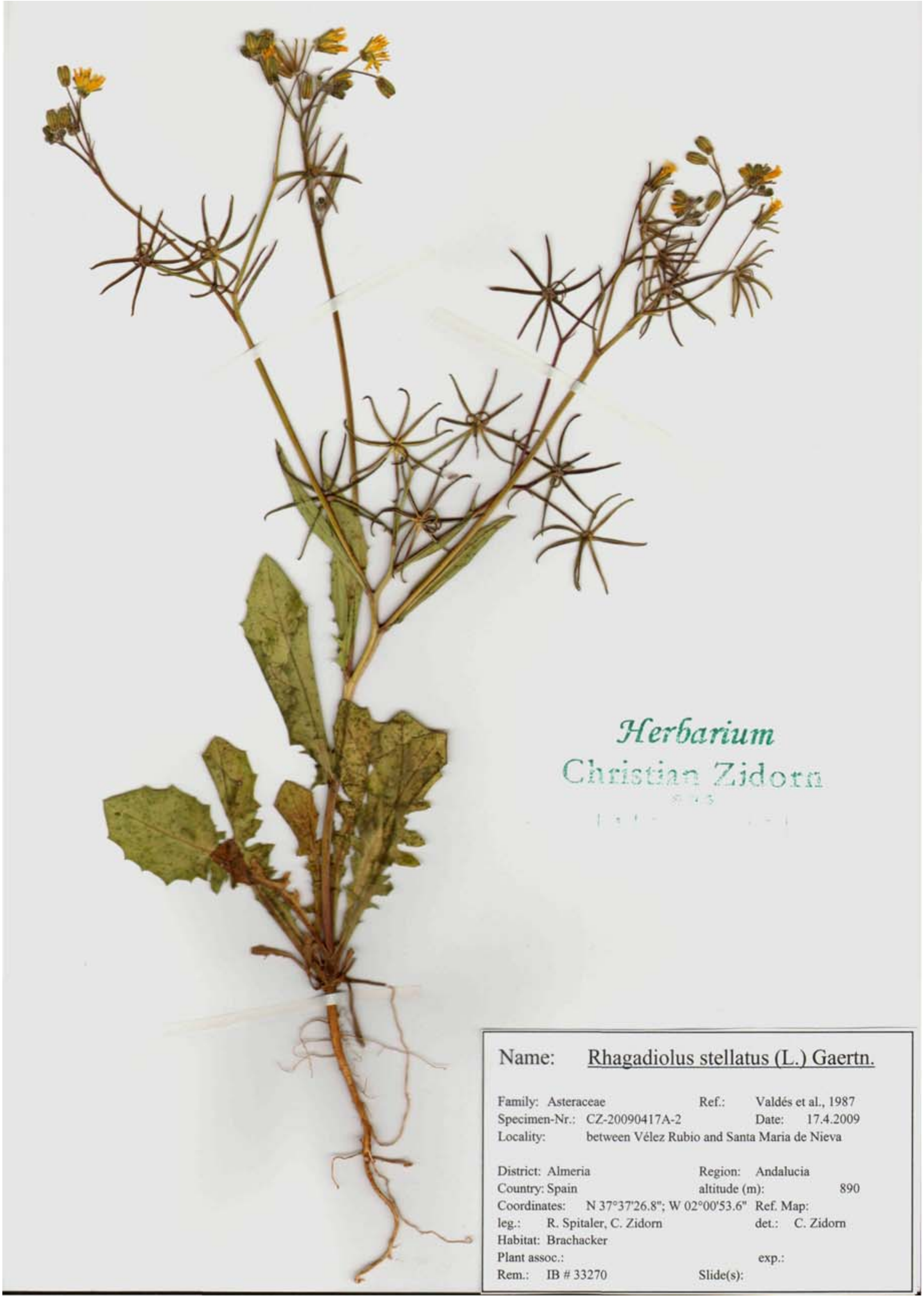

Fig. S1. Voucher of Rhagadiolus stellatus (L.) Gaertn.
